# Nasal-spraying *Bacillus* spore probiotics for pneumonia in children with respiratory syncytial virus and bacterial co-infections: a randomized clinical trial

**DOI:** 10.1038/s43856-025-01029-9

**Published:** 2025-08-07

**Authors:** Hoa Thi Le, Thuy Thi Bich Phung, Huyen Thi Bui, Hanh Thi Hong Le, Dien Minh Tran, Nhung Hong Nguyen, Hung Tuan Phan, Vu Duy Tran, Ut Vu Pham, Nha Van Phan, Huong Thu Do, Anh Hoa Nguyen, Tung Dinh Pham, Anh Thi Van Nguyen

**Affiliations:** 1 Center for Pulmonology and Respiratory Care, Vietnam National Children’s Hospital, Hanoi, Vietnam; 2 Department of Molecular Biology for Infectious Diseases, Vietnam National Children’s Hospital, Hanoi, Vietnam; 3 Spobiotic Research Center, ANABIO R&D Ltd, Hanoi, Vietnam; 4 Department of Surgical Intensive Care Unit, Vietnam National Children’s Hospital, Hanoi, Vietnam; 5 LiveSpo Pharma Ltd, Hanoi, Vietnam; 6 Faculty of Mathematics - Mechanics - Informatics, VNU University of Science, Vietnam National University, Hanoi, Vietnam

**Keywords:** Paediatric research, Viral infection, Randomized controlled trials, Microbiome, Interleukins

## Abstract

**Background:**

Our study addresses the pressing need for safe and effective treatments for pneumonia in young children caused by respiratory syncytial virus (RSV) and bacterial co-infections. This issue is particularly urgent given the absence of targeted RSV therapies and the growing threat of antibiotic resistance associated with managing bacterial co-infections.

**Methods:**

We conducted a double-blind, randomized clinical trial (ClinicalTrials.gov: NCT05929599) at Vietnam National Children’s Hospital to evaluate nasal-spraying *Bacillus* spore probiotics (LiveSpo Navax containing *B. subtilis* ANA4 and *B. clausii* ANA39 at ≥1 billion CFU/mL) in children aged 1-24 months with pneumonia due to RSV and bacterial co-infection. Participants were randomly assigned in a 1:1 ratio to receive standard care plus either LiveSpo Navax or physiological saline solution, using a simple sealed-number draw at enrollment. Primary outcomes were the median duration required to resolve common pneumonia symptoms, duration of oxygen therapy, and total treatment days.

**Results:**

A total of 120 children are enrolled (60 per group). In the final analysis, 50 participants in the Control group and 51 in the Navax group are included. The trial is completed with no serious adverse events or treatment-related side effects in either group. Navax treatment shortens the duration of eight symptoms associated with RSV pneumonia by one day, oxygen therapy by two days, and overall treatment by one day.

**Conclusions:**

The nasal-spraying *Bacillus* spore approach presents a safe, effective, and fast treatment for young children with pneumonia due to RSV and bacterial co-infections, making it especially a promising strategy for resource-limited settings.

## Plain language summary

Pneumonia is a common lung infection caused by both respiratory syncytial virus (RSV) and harmful bacteria. While antibiotics are generally effective in treating the infection, their overuse has contributed to growing antibiotic resistance. In this study, we show that LiveSpo Navax-a probiotic spray containing billions of *Bacillus* spores (beneficial bacteria that can survive tough conditions)-is safe and helpful for children with pneumonia. Spraying LiveSpo Navax directly into the noses of children helped reduce their symptoms, RSV and bacterial infections, as well as the need for antibiotics. This affordable, simple, and fast treatment offers a promising way to support recovery from pneumonia, especially in areas with limited access to healthcare.

## Abstract (web summary)

Le, Phung, Bui et al. investigate nasal-spraying *Bacillus* spore probiotics (LiveSpo Navax) as a supportive treatment for Respiratory Syncytial Virus pneumonia with bacterial co-infections in young children. Use of the probiotic reduces symptoms, antibiotic use, viral and bacterial loads, and improves immune and nasal microbiota.

## Introduction

Respiratory syncytial virus (RSV) is a leading cause of lower respiratory tract infections (LRTIs), including pneumonia, in young children worldwide^1^. In regions with high incidences of lung infections, severe pneumonia requiring hospitalization occurs in 7–13% of cases^2,3^. RSV is responsible for up to 40% of pediatric pneumonia cases, with nearly 90% of children infected by age two, and up to 40% of these cases resulting in lower respiratory tract infections (LRTIs) during their first infection^4,5^. In 2015, RSV-associated LRTIs led to an estimated 12 million episodes, 2.3 million hospitalizations, and 43,800 deaths among infants under one year, underscoring its substantial health burden^6^. While the majority of RSV-LRTIs are solely viral, a minority are worsened by secondary co-infections with bacteria, mostly *Streptococcus pneumoniae*, *Haemophilus influenzae*, and *Moraxella catarrhalis*^7–9^. In the case of RSV bronchopulmonary infection, the rate of these bacteria found in collected samples ranges from 26.3% to 43.6%, identified through cultures from respiratory specimens, such as sputum and endotracheal aspirates, as well as blood cultures^10,11^. Bacterial co-infection in children with severe RSV bronchiolitis and pneumonia substantially increases clinical disease severity, such as an increase in the need for mechanical ventilation, prolonged hospital stays, and a higher mortality rate of up to 20% in cases requiring intensive care^12–15^.

Currently, while there is no vaccine or specific treatment for pediatric RSV infection, two preventive strategies are available globally^16,17^. One option is maternal vaccination, which enables the transfer of protective antibodies from mothers to infants through the placenta, providing temporary immunity during the highest-risk period of infancy^18^. The second strategy is the use of monoclonal antibodies, such as nirsevimab, which has demonstrated notable efficacy in reducing RSV infections and hospitalizations^16^. However, access to these interventions remains limited in low-income regions, where RSV-related mortality is the highest. Additionally, alternative options, such as the monthly administration of palivizumab, are too costly for widespread use^17^.

The treatment of RSV-related pneumonia follows the general protocol. Despite the availability of the aerosolized antiviral drug ribavirin for the treatment of RSV bronchiolitis/LRTI, the drug is expensive, risky for children, and has not been approved for the treatment of pneumonia^19,20^. Once patients with RSV infection experience respiratory failure, oxygen therapy is typically prescribed^21^. In RSV cases with bacterial co-infections, patients often require the use of antibiotics, with potential side effects and threatening development of resistance^22,23^.

Probiotics have recently gained attention as a safe and promising option for the prevention and treatment of respiratory infections. According to the FAO/WHO, probiotics are live microorganisms that provide health benefits to the host when taken in adequate amounts^24,25^. Numerous clinical trials have demonstrated the efficacy of oral probiotics containing *Lactobacillus rhamnosus* GG, *Bacillus subtilis* DE111, *B. coagulans* GBI-30, and *B. animalis* subsp. *lactis* Bl12 in reducing symptoms and preventing respiratory tract infections^26–29^. However, these studies primarily focused on either prevention or treatment of mild cases of RTIs, and the benefit of using probiotics for ARTIs, such as RSV pneumonia, remains largely unexplored. Importantly, orally ingested probiotics do not offer immediate benefits due to the time required for modulation of the immune response^26–28^. In contrast, alternative delivery methods, such as nasal sprays, may provide more localized and immediate effects on the upper respiratory tract, directly influencing nasal microbiota and mucosal immunity. Our recent study revealed that nasal-spraying *Bacillus* spore probiotics (LiveSpo Navax, a physiological saline solution containing ≥1 billion/mL of highly purified spores of two bacterial strains *B. subtilis* ANA4 and *B. clausii* ANA39) promptly and effectively alleviate symptoms of acute respiratory infections (ARTIs) caused by RSV and influenza virus infections^30,31^. While the previous study demonstrated that administering probiotics via nasal spray could provide a rapid and effective symptomatic treatment for ARTIs, the scope of the trial was limited to upper respiratory infections (URTIs), which do not require oxygen therapy^30,31^. In the current study, we conducted a double-blind, randomized, and controlled clinical trial examining the efficacy of LiveSpo Navax in supporting the treatment of young children with pneumonia caused by RSV and bacterial co-infection. This group of patients with severe cases of LRTIs who require oxygen therapy represents a critical unmet need in the treatment of RSV-related pneumonia. We hypothesized that by reducing the viral load and co-infection bacterial levels in the upper respiratory tract, LiveSpo Navax can prevent or lessen the severity of infections in the lower respiratory tract. We monitored pneumonia symptoms, antibiotic use, and changes in cytokine and IgA levels, and nasal microbiota to assess the impact of nasal-spraying *Bacillus* spore probiotics. Our findings show that LiveSpo Navax reduces the duration of eight RSV pneumonia symptoms, oxygen therapy, overall treatment time, and antibiotic use. It also lowers RSV and co-infecting bacterial loads while improving nasal immune responses and microbiota composition in these young patients.

## Methods

### Study design and ethics approval

This was a double-blind, randomized, controlled clinical trial conducted at the Center for Pulmonology and Respiratory Care, Vietnam National Children’s Hospital, from July 2023 to July 2024. Patient enrollment was conducted from July 2023 to March 2024. The study evaluated the efficacy of LiveSpo Navax, a nasal-spraying probiotic, in addition to standard care, compared to a placebo (physiological saline solution), in young children hospitalized with RSV-induced pneumonia and bacterial co-infections. Ethics approval was obtained from the Ethics Committee in Medical Research of the Vietnam National Children’s Hospital under Decision No.1241/BVNTW-HDDD on May 23, 2023. The research was conducted in accordance with the ethical principles outlined in the Helsinki statement, the ICH GCP guidelines, and the prevailing ethical regulations and standards established by the Vietnam Ministry of Health for research involving human subjects. Comprehensive information about the research was provided to all parents of pediatric patients who volunteered for the study, and their consents were obtained through the necessary forms. The study was registered with ClinicalTrials.gov, US National Library of Medicine (Identifier No: NCT05929599) on 30/06/2023.

### Intervention product

Nasal-spraying probiotics LiveSpo Navax (LiveSpo Pharma, Hanoi, Vietnam) was formulated as a physiological saline solution containing *Bacillus subtilis* ANA4 (accession no. MT123906.1 in NCBI) and *B. clausii* ANA39 (accession no. MT275656.1 in NCBI) spores at a concentration exceeding 1 × 10^9^ CFU/mL^30,31^. LiveSpo Navax was manufactured as a Class-A medical device product (No: 210001337/PCBA-HN), compliant with ISO 13485:2016 standards and approved by the Hanoi Health Department, Ministry of Health, Vietnam. The taste and smell of LiveSpo Navax (intervention product) were indistinguishable from those of physiological saline solution (control product). Due to the opaque plastic container, the color and turbidity of the LiveSpo Navax suspension were unidentifiable.

### Participants, sample size, and randomisation

The inclusion criteria for this study were: (i) children (male/female) aged from 1 to 24 months; (ii) hospitalization due to pneumonia according to the WHO criteria^32^ (iii) positive RSV rapid test; (iv) confirmed bacterial co-infection by real-time PCR assay; (v) consent from the pediatric patient’s parents to participate in the study, including their understanding and signing of the research consent form.

The exclusion criteria were: (i) children with underlying medical conditions such as congenital heart disease or airway malformation; (ii) hospital-acquired pneumonia; (iii) newborn babies; (iv) history of drug allergy; (v) discharge before day 3; (vi) loss to follow-up or withdrawal from the trial; (vii) participants continuing in the trial but with missing data; and (viii) caregivers meeting criteria for psychiatric disorders other than depression and/or anxiety to ensure the accuracy and reliability of baseline data and caregiver-reported observations.

Both patients requiring and not requiring oxygen therapy were included in this study. Requirement for oxygen therapy was recorded as a baseline characteristic.

Based on the clinical design and data obtained in our prior study^30,31^, patient cohort size (*n* = 49 per group) of this study was determined based on the assumption that LiveSpo Navax treatment alleviates pneumonia symptoms resulting from RSV and bacterial co-infection, including rhinitis, fever, retractions, dry rales, moist rales, by 25% more than the Control group (standard care), with a significance level of alpha = 0.05 and a power level of 0.85^30,31^. Initially, 285 children with respiratory infections due to RSV were screened for eligibility, and 120 eligible participants (*n* = 60 per group) were randomly assigned using a lottery system to the Control and Navax groups, anticipating a potential 20% patient dropout during follow-up treatment.

**Fig. 1:**
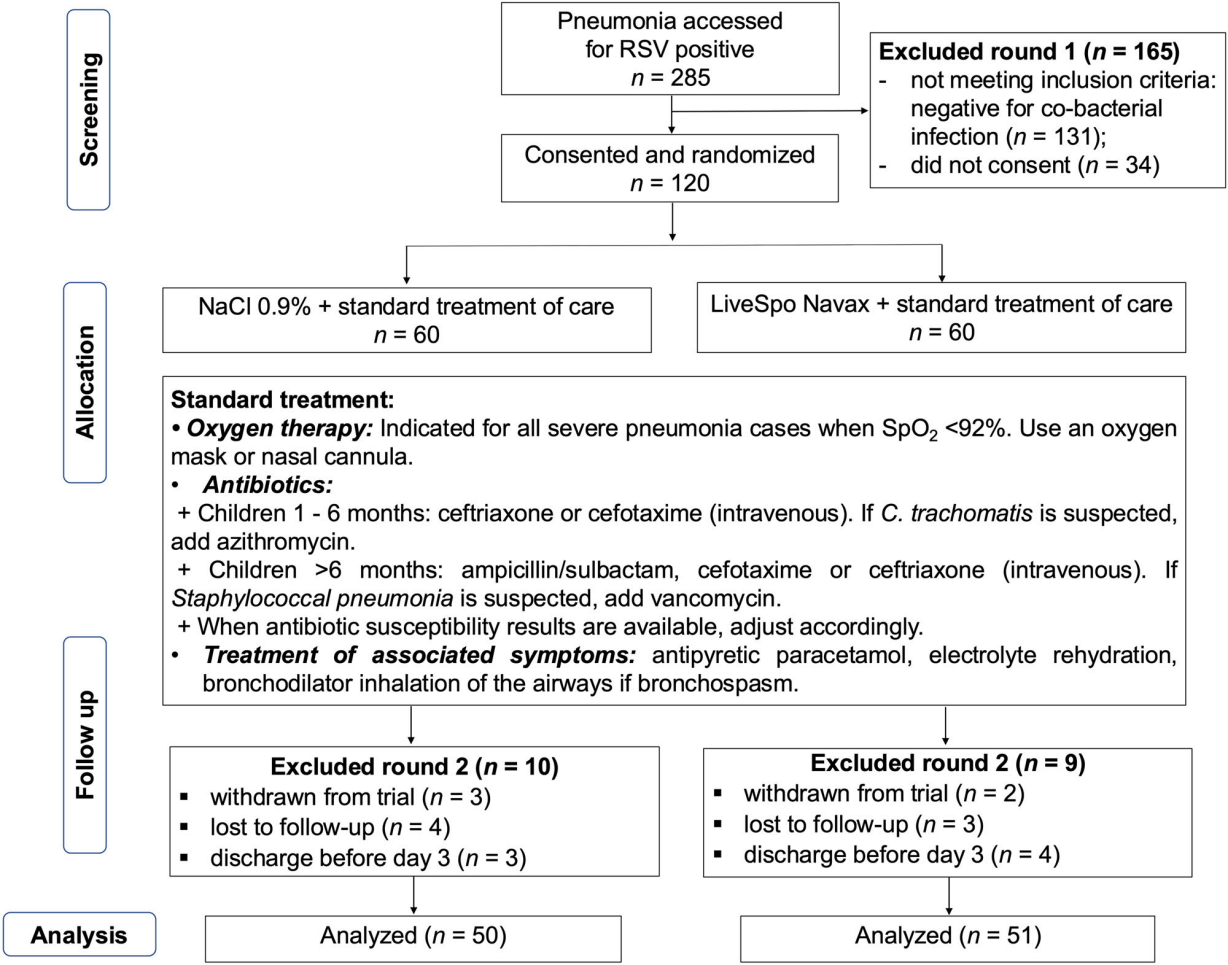
CONSORT diagram. Flowchart illustrating the design and data analysis of the NCT05929599 clinical trial conducted from July 2023 to July 2024.

Participants were randomly allocated to either the control group (referred to as the Control group) receiving 0.9% NaCl, or the experimental group (referred to as the Navax group) receiving the probiotics LiveSpo Navax. Randomisation was conducted in a 1:1 ratio using simple randomisation by lottery draw upon obtaining informed consent from the children’s parents. The chief nurse randomly selected sealed-paper coded numbers 1 or 2 from a carton box containing equal numbers of each code and immediately assigned these numbers to the participants. Product bottles were labeled with codes plus patient ID, along with patient name, date of birth, and room number to avoid confusion. Confidentiality was maintained throughout the study. Group allocations remained blinded to all personnel involved in patient care, treatment administration, and laboratory analyses. Operational adjustments were managed using anonymized codes, ensuring that the double-blind design was strictly preserved. The study design’s flowchart is presented in Fig. 1.

### Questionnaires, treatment procedures, and clinical observation

The parents of the patients were required to provide their children’s information. Nurses were provided with coded sprayers containing blinded samples and trained to administer the sprays as follows: 3 sprays per nostril (total of 6 sprays for both nostrils) and 3 sprays into the throat per dose. Each spray delivered approximately 50 μL of physiological saline solution, with or without 2.5 × 10⁸ *Bacillus* spores. This procedure was conducted three times daily, in the morning (at 8–9:00 a.m.), afternoon (3–4:00 p.m.), and evening (at 8–9:00 p.m.), and continued until hospital discharge. These nasal spray products were used concurrently with routine hospital treatment as indicated in Fig. 1. Antibiotic susceptibility tests were conducted for patients testing positive for bacterial co-infection, and suitable antibiotic medications were prescribed accordingly.

Throughout the treatment period, patients were daily monitored for typical clinical features associated with RSV-induced pneumonia with bacterial co-infection, following routine clinical examination methods approved by the Vietnam National Children’s Hospital, under the WHO guidelines^33^. Clinical status included symptoms such as cough, wheezing, fever, rhinitis, retractions, dry and moist rales, diarrhea, and vomiting, along with vital signs like heart rate (beats/min), respiratory rate (breaths/min), and oxygen saturation (SpO_2_). Treatment days are defined as the time taken for a patient to fully recover from a specific symptom, marked by the first symptom-free day during treatment. Median treatment days between the two groups were compared using the Mann-Whitney test.

### Outcomes and safety assessments

The primary outcomes included the median treatment duration required to alleviate typical clinical symptoms of pneumonia (rhinitis, fever, retractions, dry rales, moist rales, wheezy, cough, diarrhea, vomiting), duration of oxygen therapy, and total treatment days.

The secondary outcomes were fold reduction of RSV infection and bacterial co-infections (*S. pneumoniae*, *H. influenzae*) at day 3 compared to day 0; nasal cytokine levels (IL-6, IL-8, TNF-α) and IgA levels at day 3 compared to day 0; nasal microbiota composition at day 3 compared to day 0.

Safety was assessed by monitoring for focal bacterial infections, nasal irritation or discomfort during administration, allergic reactions, systemic side effects such as nausea or vomiting, and daily vital signs (heart rate, respiratory rate, SpO₂) throughout the treatment period.

### Routine diagnostics at hospital

Screening of RSV-infected cases from nasopharyngeal samples at day 0 was initially conducted by using the BD Veritor System for rapid detection of the RSV kit (Becton Dickinson, NJ, US). Detection of respiratory bacterial co-infection from nasopharyngeal samples was performed through microbial culture assays on specific media. Both tests were subsequently confirmed by Real-time RT/PCR. At day 0, nasopharyngeal swabs were collected by taking two separate swabs from the nostrils of each child. These swabs were placed in sterile plastic tubes and transported to two diagnostic departments at the Vietnam National Children’s Hospital within 30 min of collection. One swab was treated with 1 mL of 0.9% saline (no antibiotics) for microbial culture to identify bacterial co-infections, ensuring uninhibited bacterial growth. Another swab was treated with 1 mL of Universal Transport Medium (UTM, Copan Diagnostics Inc., CA, USA) and used for molecular and immunological assays as detailed below. At day 3, a single nasopharyngeal swab was collected for molecular and immunological assays.

To determine inflammation as an expression of a bacterial infection, CRP concentrations and white blood cell counts were measured using blood samples. A chest X-ray was performed for visualization of lung consolidation, ground-glass opacity, perihilar opacities, hyperinflation, and atelectasis. These three tests were conducted only at day 0, following the standard procedures at Vietnam National Children’s Hospital due to ethical concerns aimed at minimizing unnecessary invasive blood sampling and radiation exposure. This approach reduced discomfort, ensured patient safety, and provided sufficient sub-clinical data for the study.

### Real-time PCR for detecting microorganisms in nasopharyngeal samples

The protocols for RSV and bacterial co-infection detection were standardized according to ISO 15189:2022 criteria, routinely employed for clinical sample testing in the Department of Molecular Biology for Infectious Diseases, Vietnam National Children’s Hospital. In brief, DNA/RNA was extracted from 200 µL nasopharyngeal samples (repeated twice) using the MagNA Pure LC Total Nucleic Acid Isolation Kit (Roche Diagnostics, IA, US) automatic sample extraction system according to the manufacturer’s instructions. Then, 100 µL of the purified DNA/RNA was divided into three PCR tubes (approximately 30 µl/tube) and stored at −80 °C. Each tube of −80 °C purified DNA/RNA was thawed only once to obtain 5 µl, serving as the template for individual real-time PCR/RT-PCR tests to detect RSV, bacterial co-infections, and probiotic germs (*B. subtilis* and *B. clausii*).

Semi-quantitative assays for measuring changes in RSV load and co-infection bacterial concentrations in nasal tract between days 0 and day 3 were conducted using the real-time RT/PCR method employing the CFX 96Dx instrument (Bio-Rad Laboratories, CA, USA).

Detection of RSV was conducted following a previous study^30,31^. In brief, real-time RT-PCR reactions included initial reverse transcription at 50 °C for 30 min, followed by amplification and detection cycles at 95 °C for 15 s and 60 °C for 1 min. Simultaneous detection of multiple bacterial co-infections utilized the Allplex Respiratory Panel 4 kit (Seegene, Seoul, Korea), following methodologies from prior studies^30,31^. This kit identifies seven pathogenic bacterial species, including *Bordetella parapertussis* (BPP), *Bordetella pertussis* (BP), *Chlamydophila pneumoniae* (CP), *Haemophilus influenzae* (HI), *Legionella pneumophila* (LP), *Mycoplasma pneumoniae* (MP), and *Streptococcus pneumoniae* (SP). Other pathogens, such as *Moraxella catarrhalis* and *Staphylococcus aureus* were not included in real-time PCR screening due to absence of ISO-compliant diagnostic protocols for these bacteria. The reactions followed the company’s protocol: an initial denaturation at 95 °C for 15 min, followed by amplification and detection cycles at 95 °C for 10 s, 60 °C for 1 min, 72 °C for 10 s. The real-time PCR assays were completed within 5 h to ensure timely randomisation of patients into study groups and administration of nasal spray interventions on the same day following admission to the treatment department.

Standardisation for the analysis of RSV and bacterial co-infections required adjustments to a *C*_*t*_ of < 40 to confirm their positivity. The reduction levels (2^△*Ct*^) of RSV load and bacterial co-infection concentrations were determined as the difference between *C*_*t*_ (threshold cycle) values. △*C*_*t*_ for target genes was calculated as *C*_*t*_ at day 3 - *C*_*t*_ at day 0, ensuring equal adjustment of the internal control *C*_*t*_ across all samples. Comparisons of 2^Δ*Ct*^ between the two groups were made using the Mann-Whitney test. Multiple logistic regression was applied to create scatter plots visualizing the association between reductions in RSV load and bacterial co-infection concentrations.

Detection of *B. subtilis* ANA4 and *B. clausii* ANA39 in nasopharyngeal samples was performed at day 0 and day 3 using real-time PCR SYBR Green. Specific primers designed for detecting *B. subtilis* and *B. clausii* were employed under the following conditions: 95 °C for 10 min, amplification for 45 cycles at 95 °C for 15 s, 60 °C for 20 s, 72 °C for 30 s. The read-out standardisation for the analysis of *B. subtilis* and *B. clausii* was set at *C*_*t*_ < 40 to confirm true positivity. The protocol for detecting *B. subtilis* and *B. clausii* was developed following the ISO 17025:2017 guidelines and was specifically applied for research purposes within the Department of Molecular Biology for Infectious Diseases at the Vietnam National Children’s Hospital.

### ELISA assays for cytokine and IgA levels

Other secondary outcomes including (i) pro-inflammatory cytokines levels (pg/mL): interleukin (IL-6, IL-8) and TNF-α; and (ii) Immunoglobulin A (IgA) levels (mg/mL) in nasopharyngeal samples at days 0 and 3 were quantified using an enzyme-linked immunosorbent assay kit (ELISA) following to the manufacturer’s instructions. IL-6 and TNF-α were quantified from 100 µL samples by the IL-6 Human ELISA and TNF-α Human ELISA kit, respectively (Invitrogen/Thermo Fisher Scientific, MA, US). IL-8 concentration was assessed by 100 µL samples by Human IL-8 DuoSet ELISA kit (R&D Systems, MN, US). IgA level was measured by 10 µL samples by Human IgA ELISA kit (Invitrogen/Thermo Fisher Scientific, MA, US). Samples were measured by the SpectraMax Plus384 Microplate Reader system. The results were analysed by using SoftMax Pro 6.3 Software (Molecular Devices, CA, US). The concentrations of IL-6, IL-8, TNF-α (ng/mL), and IgA (µg/mL) in the nasopharynx were determined using standard curves generated with the calibrators provided in the respective kits, adhering strictly to the manufacturers’ protocols. The Wilcoxon test was used to calculate the median differences in IL-6, IL-8, TNF-α, and IgA levels at day 0 and day 3 within each group. Comparisons of cytokine concentrations between the two groups were made using the Mann-Whitney test. Multiple logistic regression was applied to create scatter plots visualizing the association between reductions in RSV load and changes (Δ) in IL-6, IL-8, TNF-α, and IgA levels at day 3 compared to day 0.

### 16S rRNA Metagenomic analysis of nasal microbiota in nasopharyngeal samples

Nasopharyngeal samples were collected from children with pneumonia caused by RSV and bacterial co-infections treated at the Center for Pulmonology and Respiratory Care at the Vietnam National Children’s Hospital for 16S rRNA metagenome analysis. Sixteen nasopharyngeal fluid samples from each group at both day 0 and day 3 that guaranteed quality and met the selection criteria were selected for NGS analysis. The nasopharyngeal samples were randomly selected in a stratified manner to ensure similar indices regarding age, sex, weight, pre-hospital illness, and median reduction in RSV after 3 days. This approach was used to obtain data reflecting the microbiota of representative participants in this study. Total DNA was extracted using a DNAeasy Mini Kit (Qiagen, Stockach, Germany). The V3–V4 hypervariable region of the 16S rRNA gene was amplified using the Herculase II Fusion DNA Polymerase Nextera XT Index Kit V2, and the 300 bp paired-end DNA libraries were constructed using the 16S Metagenomic Sequencing Library Preparation kit. The 16S rRNA library sequencing was performed by Macrogene Inc. (Seoul, Republic of Korea) on an Illumina MiSeq platform (Illumina, San Diego, CA, USA), and the raw data were quality checked and base calling was conducted using Real Time Analysis (RTA). The CD-HIT-out-Miseq package was used to trim the fastq reads, filter out the short reads, and remove noise sequences^34^. Ambiguous and chimeric reads were identified and removed by rDnaTools (PacBio, USA). Remaining reads were clustered into operational taxonomic units (OTUs) using a greedy algorithm at a cut-off level of 97% sequence similarity. Qiime version 1.9.1.1 package was used for diversity analysis, including OTUs abundance and alpha rarefaction, taxonomy diversity, and beta diversity^35^. The minimum number of reads for each group was 50,000 and QV20 quality scores exceeded 99%, ensuring robustness for subsequent sequence analysis. All bioinformatics work was performed by the technical service of Macrogene Inc. (Seoul, Republic of Korea). Alpha diversities within and between study groups were assessed during various visits. Four metrics of alpha diversity were analyzed: (1) the Chao 1 index, (2) the Shannon diversity index, which considers species richness and abundance, (3) Pielou Evenness (a measure of microbial abundance distribution), and (4) Faith’s PD (species conservation). The Wilcoxon test and Mann–Whitney test were used for comparisons of alpha diversity within each group and between the two groups. Beta diversity was assessed using Bray-Curtis dissimilarity, and statistical significance was determined using Permutational Multivariate Analysis of Variance (PERMANOVA). Further microbiota analysis visualization and characteristic marker analysis, including distribution of phyla, genera, and species were conducted using the R package microbiome^36,37^. Lefse algorithm analysis, incorporating statistical significance tests (Kruskal and Wilcoxon), was conducted to evaluate significant differences in the distribution of genera and species between groups. The abundance difference for a given bacterial genus or species between the Control and Navax groups was quantified as the log_2_ fold change (LFC) in counts for the corresponding OTU, calculated as log_2_(Control/Navax).

### Statistics and reproducibility

Individual medical records and patient’s information were collected and compiled into a dataset. Tabular analysis was conducted on dichotomous variables using either the χ^2^ test or Fisher’s exact test when the expected value of any cell was below five. Depending on whether the data distribution is normal or non-normal, either the *t* test, Wilcoxon test, or the Mann–Whitney test (two-tailed) was used to compare the medians of quantitative variables within the same group or between two groups. Multiple logistic regression was used to generate scatter plots for visualization. Differences in two-dimensional distributions between groups were confirmed using the non-parametric energy test. GraphPad Prism v8.4.3 (GraphPad Software, CA, US) was used for statistical and graphical analyses of all primary outcomes and most secondary outcomes, including RSV load, co-infecting bacterial concentrations, and cytokine and IgA levels. Nasal microbiota composition was analysed using the R microbiome package. All analyses were conducted with a significance level of *p* < 0.05.

Sample sizes used for analysis were *n* = 50 (Control group) and *n* = 51 (Navax group) for clinical symptoms, vital signs, RSV and co-bacterial loads, and IL-6 and TNF-α cytokine levels. For IL-8 cytokine and IgA levels, samples from 50 patients in each group were analyzed. Nasal microbiota analysis was conducted on 16 samples per group. Each sample represents a biologically independent patient. No technical replicates were performed.

### Reporting summary

Further information on research design is available in the Nature Portfolio Reporting Summary linked to this article.

## Results

### Trial design and participant’s baseline characteristics

Among 285 participants screened for eligibility, 120 patients were randomized equally (1:1) into two groups: the Control group (*n* = 60), which received physiological saline solution plus standard treatment of care, and the Navax group (*n* = 60), which received LiveSpo Navax in addition to standard treatment of care. Each group received 3 sprays of either control 0.9% NaCl or LiveSpo Navax in each nostril and throat, totaling 9 sprays per time meaning 27 sprays per day (administered 3 times daily). This dosing regimen was initiated about 5 hours after hospital admission and continued until discharge, typically lasting 7–10 days. By the end of the treatment period, 50 patients in the Control group and 51 patients in the Navax group were included in the final analysis (Fig. 1).

Patients’ demographic characteristics were shown in Table 1. All children with pneumonia due to RSV infection and bacterial co-infection were under 2 years old (1 to 24 months), with the majority being under 6 months old (>58%). Co-infection was defined as a condition where RSV patients were also confirmed with the presence of any type of bacteria based on RT-PCR assay. There were no significant differences between the two groups regarding (i) age (*p* = 0.9331), (ii) gender distribution (*p* = 0.9331), (iii) weight (*p* = 0.3195**)**, and (iv) days of illness before treatment (*p* = 0.1489). Patients hospitalized with pneumonia exhibited various clinical symptoms, such as cough, wheeze, fever, rhinitis, retractions, dry and moist rales, diarrhea, and vomiting. Physical signs included poor feeding (*p* = 0.4364), excessive crying (*p* = 0.7152), convulsions (*p* = 0.6779), and cyanosis (*p* = 0.1894), alongside abnormal vital signs such as tachycardia (beats/min) (*p* = 0.4906), tachypnea (breaths/min) (*p* = 0.625**)**, and low oxygen saturation (SpO_2_) (*p* = 0.2575). Before treatment, no significant difference between the two groups was observed.

**Table 1:** Demographic characteristics of pneumoniae children before treatment

Characteristic	Control group (*N* = 50)	Navax group (*N* = 51)	*p* value
***Age (months)**** n* (%)			
≤6	29 (58.00)	30 (58.82)	0.9331^a^
>6	21 (42.00)	21 (41.18)	
***Gender**** n* (%)			
Male	29 (58.00)	30 (58.82)	0.9331^a^
Female	21 (42.00)	21 (41.18)	
***Weight (Mean ± SD)***	7.474 ± 2.001	7.214 ± 2.139	0.3195^b^
***Days of sickness before treatment (Mean ± SD)***	3.36 ± 1.336	3.67 ± 1.465	0.1489^b^
***Physical symptoms**** n* (%)			
Poor feeding	46 (92.00)	49 (96.00)	0.4364^c^
Excessive crying	46 (92.00)	48 (94.12)	0.7152^c^
Convulsion	2 (4.00)	4 (7.84)	0.6779^c^
Cyanosis	31 (62.00)	25 (49.02)	0.1894^c^
***SpO***_***2***_ ***(%)****, n* (%)			
≥92%.	9 (18.00)	14 (27.45)	0.2575^a^
<92%	41 (82.00)	37 (72.55)	
Tachycardia (beats/min) *n* (%)	27 (54.00)	31 (60.78)	0.4906^a^
Tachypnea (breaths/min) *n* (%)	27 (54.00)	30 (58.82)	0.625^a^

Bold and Italic text represents subgroup means.

^a^Chi-Square test.

^b^Mann–Whitney test.

^c^Fisher’s Exact test.

Additionally, baseline sub-clinical characteristics, including chest X-rays (*p* = 0.9639), hematology and biochemistry (*p* = 0.2661, 0.423, and 0.4255), serum C-reactive protein (CRP) levels (*p* = 0.1339), number of cases positive for RSV detected by rapid test and real-time PCR assay (>0.9999), the median *C*_*t*_ values for RSV detection (24.45 in the Control group and 23.20 in the Navax group), and number of cases of bacterial co-infection determined by real-time PCR assay (*p* = 0.1258) and microbial culture (*p* = 0.8309), showed no notable variation between the two groups before treatment (Table 2 and Supplementary Table 1).

**Table 2:** Sub-clinical characteristics of pneumoniae in children before treatment

Characteristic	Control group (*N* = 50)		Navax group (*N* = 51)		*p* value
	Total. *n* (%)	Min - max	Total. *n* (%)	Min - max	
**Chest X-ray**					
Normal	1 (2.00)		1 (1.96)		0.9639^a^
Ground-glass opacity nodules in both lung fields	39 (78.00)		37 (72.55)		
Bronchial wall thickening	2 (4.00)		3 (5.88)		
Upper lobe opacities P	3 (6.00)		3 (5.88)		
Osler's nodes, interstitial injury, hyperinflation	5 (10.00)		7 (13.73)		
**RSV Positive**					
Rapid test	50 (100)		51 (100)		>0.9999^a^
Real-time PCR	50 (100)		51 (100)		>0.9999^a^
**Hematology and Biochemistry**					
***Total white blood cells (G/L)*** -^b^		4.99–26.96		5.69–25.50	
Below standard range	0 (0.00)		0 (0.00)		-^c^
Within standard range	45 (90.00)		42 (82.35)		0.2661^b^
Above standard range	5 (10.00)		9 (17.65)		
***Neutrophil (G/L)*** -^c^		0.9–19.36		1.36–22.80	
Below standard range	1 (2.00)		0 (0.00)		0.423^a^
Within standard range	35 (70.00)		40 (78.43)		
Above standard range	14 (28.00)		11 (21.57)		
***Hemoglobin (g/dL)*** (Children ≥ 6 months)		89–125		86–138	
<110	8 (16.00)		5 (9.80)		0.4255^b^
≥110	15 (30.00)		16 (31.37)		
***Total platelet count (G/L)***					
<150	0 (0.00)		0 (0.00)		-^c^
150 - 450	34 (68.00)		32 (62.75)		0.579^b^
>450	16 (32.00)		19 (37.25)		
**CRP (mg/L)**					
>40	36 (72.00)		43 (84.31)		0.1339^b^
≤40	14 (28.00)		8 (15.69)		
**Bacterial co-infection detected by real-time PCR**					
*H. influenzae (HI)*	33 (66.00)		25 (49.02)		0.1258^a^
*S. pneumoniae (SP)*	5 (10.00)		11 (21.57)		
*M. pneumoniae (MP)*	2 (4.00)		1 (1.96)		
*B. pertussis (BP)*	0 (0.00)		1 (1.96)		
*H. influenzae + S. pneumoniae*	7 (14.00)		13 (25.49)		
*H. influenzae + B. pertussis*	1 (2.00)		0 (0.00)		
*S. pneumoniae + M. pneumoniae*	1 (2.00)		0 (0.00)		
*H. influenzae + S. pneumoniae + M. pneumoniae*	1 (2.00)		0 (0.00)		

Bold and Italic text represents subgroup means.

^a^Chi-Square test.

^b^Fisher’s Exact test.

^c^There is no remaining patients to compare the significance.

It is important to note that real-time PCR detected *H. influenzae* (HI) and *S. pneumoniae* (SP) in a relatively high number of cases, while *M. pneumoniae* (MP) and *B. pertussis* (BP) were found in fewer cases (Table 2). Microbial culture, used exclusively for guiding antibiotic treatment, detected a few additional cases of *M. catarrhalis*, *S. aureus*, and *P. aeruginosa* infections, which were not among the seven bacterial species detectable by the real-time PCR kit (Supplementary Table 1). Therefore, subsequent experiments focused on evaluating changes in *H. influenzae* (HI) and *S. pneumoniae* (SP) in nasopharyngeal samples.

### Safety and symptom relief: shortening treatment duration for symptoms and antibiotic use for pneumonia in children by nasal-spraying *Bacillus* spores

During the treatment period, all participants in both groups showed no symptoms of focal bacterial infection, such as the development of new pharyngeal abscesses, severe otitis media, or worsening rhinitis. There were no cases of nasal mucosal irritation, discomfort during administration, or systemic side effects such as nausea or vomiting. Furthermore, there were no abnormalities observed in vital signs, including heart rate, respiratory rate, and SpO_2_, following 9 consecutive administrations of either probiotic or placebo sprays over 3 follow-up days (Supplementary Fig. 1). Previous studies have shown that the *Bacillus* probiotic spore nasal spray is safe for children with RSV and influenza virus^30,31^. This study confirms its safety for pediatric pneumonia patients. The efficacy of *Bacillus* spores in alleviating typical clinical symptoms of pneumonia at days 3 and 5 was then evaluated and presented in Table 3. Before treatment (day 0), baseline clinical characteristics of the participants, including cough (*p* > 0.9999), wheezing (*p* = 0.495), fever (*p* = 0.5257), rhinitis (*p* = 0.495), retractions (*p* = 0.2205), diarrhea (*p* = 0.7742), vomiting (*p* = 0.9151), dry rales (*p* = 0.1114), moist rales (*p* > 0.9999), tachycardia (*p* = 0.4906), tachypnea (*p* = 0.625), and the need for oxygen therapy (*p* = 0.2575) did not show statistically significant differences between the two groups. At day 3, the Navax group showed a significantly lower percentage of patients with several symptoms and vital signs, including cough (*p* = 0.0279), fever (*p* = 0.0125), retractions (*p* = 0.0034), diarrhea (*p* = 0.0478), dry rales (*p* = 0.0346), moist rales (*p* = 0.0496), and the need for oxygen therapy (*p* = 0.0074), and a trend toward a lower percentage of patients with rhinitis (*p* = 0.0692) and vomiting (*p* = 0.0876) compared to the Control group. By day 5, Navax treatment lowered the percentage of patients with rhinitis (*p* = 0.0478), and patients trended toward reduced rates of cough (*p* = 0.0905) and wheezing (*p* = 0.0506). After 5 days of treatment, we observed a similar proportion of patients showing improvements in the remaining clinical symptoms and vital signs in both the Control and Navax groups (Table 3).

**Table 3:** Clinical characteristics of pneumoniae children before, during and at the end of treatment

Characteristic	Control group (*N* = 50)			Navax group (*N* = 51)			*p value*		
	Before treatment	During and at the end of treatment		Before treatment	During and at the end of treatment		Before treatment	During and at the end of treatment	
	Day 0	Day 3	Day 5	Day 0	Day 3	Day 5	Day 0	Day 3	Day 5
Cough *n* (%)	50 (100)	48 (96.00)	26 (52.00)	51 (100)	41 (80.39)	18 (35.29)	>0.9999^a^	**0.0279**^a^	0.0905^b^
Wheezing *n* (%)	49 (98.00)	44 (88.00)	22 (44.00)	51 (100)	41 (80.39)	13 (25.49)	0.495^a^	0.2951^b^	0.0506^b^
Fever *n* (%)	39 (78.00)	6 (12.00)	1 (2.00)	37 (72.55)	0 (0.00)	0 (0.00)	0.5257^b^	**0.0125**^a^	0.495^a^
Rhinitis *n* (%)	49 (98.00)	45 (90.00)	21 (42.00)	51 (100)	39 (76.47)	12 (23.53)	0.495^a^	0.0692^b^	**0.0478**^b^
Retractions *n* (%)	43 (86.00)	27 (54.00)	6 (12.00)	39 (76.47)	13 (25.49)	6 (11.76)	0.2205^b^	**0.0034**^b^	0.9709^b^
Diarrhea *n* (%)	22 (44.00)	21 (42.00)	8 (16.00)	21 (41.18)	12 (23.53)	4 (7.84)	0.7742^b^	**0.0478**^b^	0.2343^a^
Vomiting *n* (%)	27 (54.00)	24 (48.00)	11 (22.00)	27 (52.94))	16 (31.37)	7 (13.73)	0.9151^b^	0.0876^b^	0.2773^b^
Dry rales *n* (%)	45 (90.00)	33 (66.00)	11 (22.00)	40 (78.43)	23 (45.1)	7 (13.73)	0.1114^b^	**0.0346**^b^	0.2773^b^
Moist rales *n* (%)	50 (100)	41 (82.00)	16 (32.00)	50 (98.04)	33 (64.71)	12 (23.53)	>0.9999^a^	**0.0496**^b^	0.3417^b^
Tachycardia *n* (%)	27 (54.00)	5 (10.00)	2 (4.00)	31 (60.78)	3 (5.88)	1 (1.96)	0.4906^a^	0.4874^b^	0.6175^b^
Tachypnea *n* (%)	27 (54.00)	1 (2.00)	0 (0.00)	30 (58.82)	4 (7.84)	0 (0.00)	0.625^a^	0.3624^b^	^_c^
Required oxygen therapy *n* (%)	41 (82.00)	21 (42)	6 (12)	37 (72.55)	9 (17.65)	2 (3.92)	0.2575^b^	**0.0074**^b^	0.1599^a^

Bold *p*-values indicate statistically significant differences (*p* < 0.05).

^a^Chi-Square test.

^b^Fisher’s Exact test.

^c^There is no remaining patients to compare the significance.

**Fig. 2:**
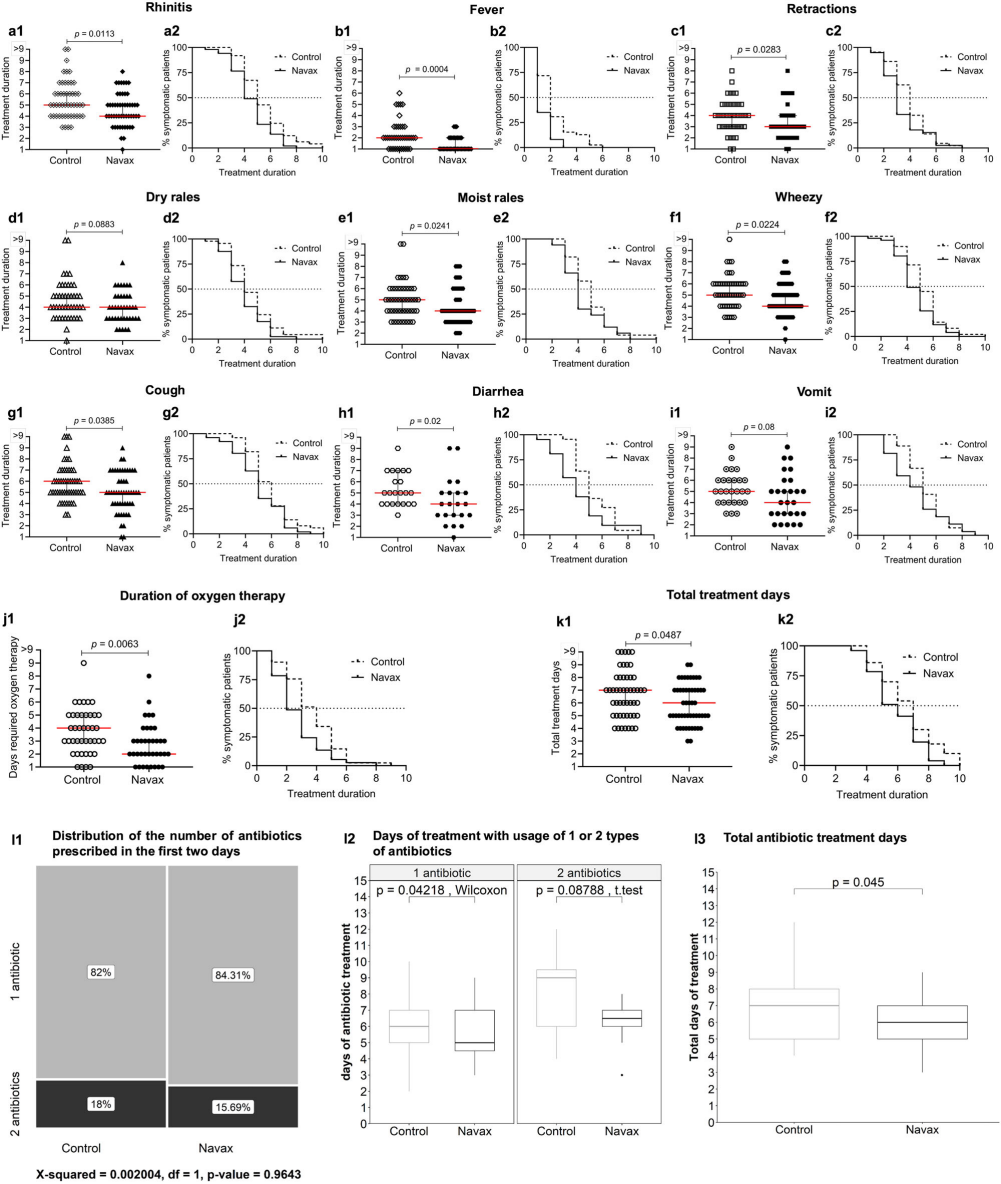
Effectiveness of *Bacillus* spore nasal spray in improving clinical RSV symptoms. Treatment duration (number of days to resolve symptoms) (**a1**–**k1**) and Kaplan–Meier analysis of estimated percentage (%) of symptomatic patients (**a2**–**k2**) observed over treatment duration for symptoms of pneumonia in the Control (dashed lines) and Navax (solid lines) groups. The distribution of the number of antibiotics prescribed in the first two days (**l1**), duration of antibiotic treatment with 1- and 2-type antibiotics (**l2**), and total antibiotic treatment days (**l3**) prescribed for children with pneumonia in the Control and Navax groups. Statistical significance was evaluated using the Mann–Whitney test. Median values with 95% CI for each group and the median difference between the two groups are shown in panels **a**–**k**. Error bars in panels **l2**–**l3** indicate the upper extreme (Q3 + 1.5 × IQR) and the lower extreme (Q1 – 1.5 × IQR). The significance level for all analyses was set at *p* < 0.05. Sample size: *n* = 50 biologically independent patients in the Control group and *n* = 51 in the Navax group.

In the trial, the primary outcome was the treatment duration required to alleviate typical clinical symptoms of pneumonia (Fig. 2a1–k1) with the time-course dependent percentage (%) of patients achieving symptom-free status serving as an alternative representation of this outcome (Fig. 2a2–k2) in the two groups. The term treatment duration refers to the number of days required to resolve a particular symptom after the first day of treatment. Notably, the Navax group experienced faster recovery, resolving most symptoms 1 day earlier compared to the Control group, including rhinitis (*p* = 0.0113; Fig. 2a1), fever (*p* = 0.0004; Fig. 2b1), retractions (*p* = 0.0283; Fig. 2c1), moist rales (*p* = 0.0241; Fig. 2e1), wheezy (*p* = 0.0224; Fig. 2f1), cough (*p* = 0.0385; Fig. 2g1), and diarrhea (*p* = 0.02; Fig. 2h1). Although the Navax group’s vomiting symptom resolved one day earlier than that of the Control group, the difference was not statistically significant (*p* = 0.08) (Fig. 2i1). These shortened recovery times improved treatment efficacy for each of those symptoms by approximately 1.2- to 2.0-fold compared to standard treatment. Nevertheless, the treatment duration for dry rales (Fig. 2d1), tachycardia, and tachypnea (Table 3) were similar between both groups. Using Kaplan-Meier analysis, we presented the percentage of symptomatic patients over the treatment period. The analyses showed that the Navax group consistently experienced lower percentages of symptomatic patients at most follow-up time points for 10 out of 11 symptoms and vital signs, as shown in Fig. 2a2–2k2. The time at which 50% of patients recovered in the Navax group was generally 1-day earlier compared to the Control group, highlighting the faster recovery facilitated by Navax treatment. For the dry rales, although there was no difference in the time to 50% recovery between the two groups, a clear trend showed a higher percentage of recovered patients in the Navax group at various follow-up time points (Fig. 2d2). Overall, LiveSpo Navax reduced the duration of oxygen therapy by 2 days (2 days in Navax vs. 4 days in Control, *p* = 0.0063; Fig. 2j1-j2) and shortened the total pneumonia treatment duration by reducing hospitalization by 1 day (6 days in Navax vs. 7 days in Control, *p* = 0.0487; Fig. 2k1-k2).

For children with pneumonia caused by RSV and bacterial co-infections, antibiotic therapy is essential to inhibit pathogenic bacteria. Antibiotics are prescribed based on standard treatment regimens and antibiogram test results, combined with close follow-up on the treatment (Table 1). The distribution of patients using 1- or 2-type antibiotic regimens, including the names and combinations of antibiotics used, was observed to be similar in the Navax and Control groups within the initial 2 days (82% in Control vs. 84.31% in Navax for 1-type antibiotic, 18% in Control vs. 15.69% in Navax for 2-type antibiotic; *p* = 0.9643) (Fig. 2l1, Supplementary Table 2). The Navax group showed a reduction in the duration of treatment with 1-type antibiotic by 1 day (*p* = 0.04218) and with 2-type antibiotics by 3 days (*p* = 0.08788) (Fig. 2l2). Overall, the total antibiotic treatment time in the Navax group was significantly reduced by 1 day (7 days in Control vs. 6 days in Navax, *p* = 0.045) (Fig. 2l3). This reduction corresponds to a 14.3% decrease in antibiotic usage for treating pneumonia.

### Reducing the concentration of RSV and co-infecting bacteria by nasal-spraying *Bacillus* spores

**Fig. 3:**
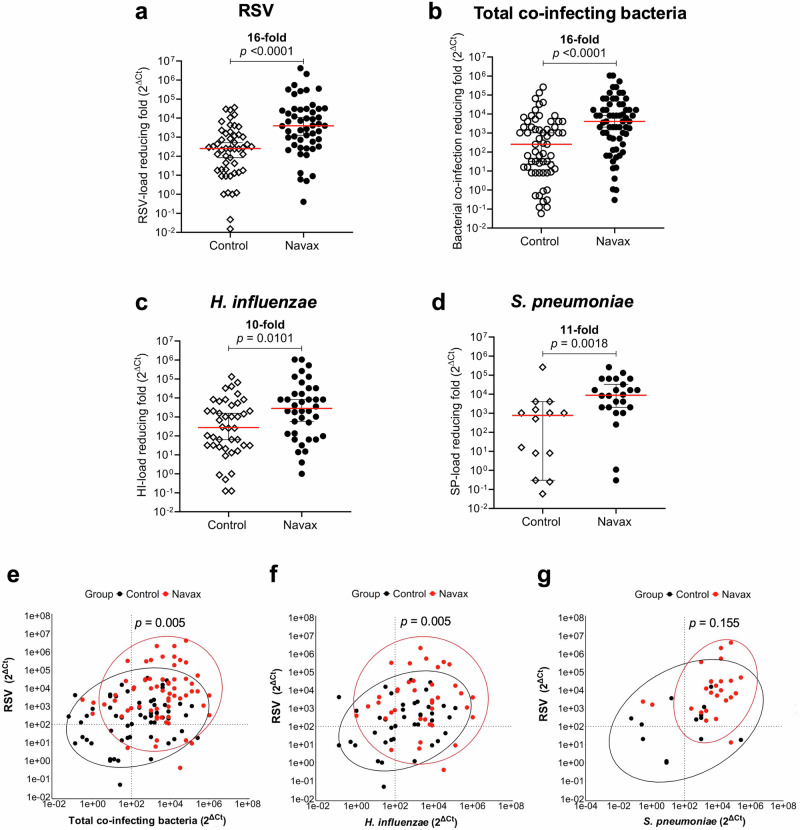
Impact of *Bacillus* spore nasal spray on viral-bacterial loads and their correlation in pediatric RSV pneumonia. Reducing-fold levels (2^△Ct^) of RSV load (**a**), total co-infecting bacterial concentration (**b**), *H. influenzae* load (**c**), and *S. pneumoniae* load (**d**) in nasopharyngeal samples of Control and Navax groups at day 3 compared to day 0. The differences in the associations between reduced RSV load and reduced total bacterial co-infection concentrations (**e**), reduced RSV load and reduced *H. influenzae* (**f**), and reduced RSV load and reduced *S. pneumoniae* (**g**) were confirmed using the energy test. The median differences for these indicators between the two groups were calculated using the Mann–Whitney test. Median values with 95% CI for each group and the median difference between the two groups are shown in panels (**a**–**d)**. All analyses were considered significant at *p* < 0.05. Sample size: *n* = 50 biologically independent samples in the Control group and *n* = 51 in the Navax group.

To explore the scientific background of how *Bacillus* spores alleviate symptoms, we conducted real-time PCR/RT-PCR TaqMan probe assay to analyse fold changes (calculated by 2^△*Ct*^, where △*C*_*t*_ = *C*_*t* day 3_ - *C*_*t* day 0_) in RSV loads and concentrations of *H. influenzae* and *S. pneumoniae* in nasopharyngeal samples, as described in the Methods section. The turnaround time for the assay is approximately 5 hours, making it well-suited for hospital operations with time constraints. Moreover, this semi-quantitative approach, certified under ISO 15189:2022, ensures reliable and standardized results. By detecting both RSV and bacteria in the same clinical sample, we confirmed that a patient was simultaneously infected with RSV and bacteria. Therefore, our analysis semi-quantified reductions in RSV loads and bacterial concentrations to primarily assess the impact of *Bacillus* spores in enhancing the effectiveness of standard therapy. As shown in Fig. 3a, the 2^△*Ct*^ data revealed a significant reduction in RSV load in the Navax group, by 3956-fold, which was 16-fold more effective than the 252-fold reduction observed in the Control group (*p* < 0.0001). Regarding the reduction in a total co-infecting bacterial concentration (including *H. influenzae*, *S. pneumoniae*, *M. pneumoniae*, *B. pertussis*), the Navax group demonstrated a substantial reduction of 4096-fold, which was 16 folds greater (*p* < 0.0001) than that in the Control group (256-fold reduction) after 3 days of treatment (Fig. 3b). Notably, among cases infected with *H. influenzae* and *S. pneumoniae*, the most common co-infecting bacterial species in respiratory tract, the reduction in *H. influenzae* and *S. pneumoniae* load was 2807 and 8801 folds, respectively, in the Navax group, which were 10–11 folds more effective than those in the Control group (*p* = 0.0101 and 0.0018, respectively; Fig. 3c, d).

Furthermore, we used multiple logistic regression to assess the link between reduced RSV load and reduced co-infecting bacterial concentrations, including *H. influenzae* and *S. pneumoniae* (Fig. 3e–g). A reduction threshold of 10² was set, reflecting the reduction levels seen in the Control group following standard hospital treatment. In the Navax group, patients with significant RSV reductions also showed substantial total co-bacterial load reductions, as seen in the red plots clustering in the upper right quadrant (Fig. 3e). This trend differed significantly from the Control group, where patients clustered around the cut-off and lower left quadrant (*p* = 0.005). Similar correlations were found between RSV reductions and reductions in individual *H. influenzae* (*p* = 0.005) and *S. pneumoniae* (*p* = 0.115) concentrations (Fig. 3f, g). *Bacillus* spores (*B. subtilis* and *B. clausii*) were detected in the Navax group’s nasopharyngeal samples on day 3, but not in the Control group, confirming proper medication intake. These results indicate that nasal-spraying *Bacillus* spores enhance the effectiveness of standard therapy by simultaneously reducing RSV and co-bacterial infection loads. It is noted that both groups received inhaled corticosteroids (budesonide) during treatment, with 41 out of 50 patients in the Control group and 41 out of 51 patients in the Navax group. This balanced use of corticosteroids minimizes potential bias in the analysis of RSV load dynamics and mucosal cytokine responses.

### Modulating cytokine and IgA responses by nasal-spraying *Bacillus* spores

**Fig. 4:**
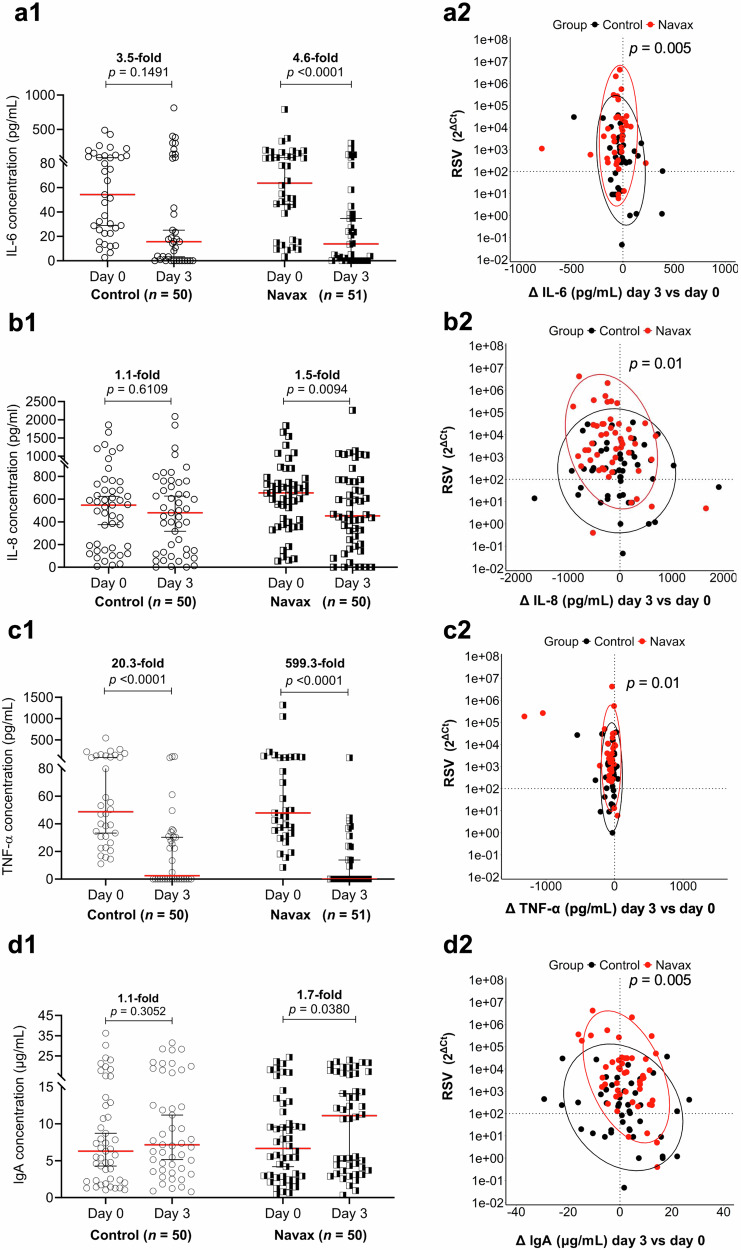
Impact of *Bacillus* spore nasal spray on pro-inflammatory cytokines and nasal-mucosal immunity. Pro-inflammatory cytokine levels (pg/mL) and IgA levels (mg/mL) (**a1**–**d1**) in nasopharyngeal samples of Control and Navax groups at day 3 compared to day 0. The association between reduced RSV load and ▵cytokine/IgA levels from day 0 to day 3 (**a2**–**d2**) was assessed using multiple logistic regression analysis. The Wilcoxon test was used to calculate the median differences in IL-6, IL-8, TNF-α, and IgA levels at day 0 and day 3 within each group. The differences in the associations between reduced RSV load and changes in IL-6 levels (**a2**), reduced RSV load and changes in IL-8 levels (**b2**), reduced RSV load and changes in TNF-α levels (**c2**), and reduced RSV loads and changes in IgA levels (**d2**) were validated using the energy test. Comparisons of cytokine concentrations between the two groups were made using the Mann-Whitney test. Only measurable samples at day 0 were included in the statistical analysis. 95% CI for the median in each group and the median difference between the two groups were shown. The significance level for all analyses was set at *p* < 0.05. Sample size: *n* = 50 biologically independent samples for all three cytokines and IgA in the Control group; *n* = 51 for IL-6 and TNF-α, and *n* = 50 for IL-8 and IgA in the Navax group.

Our previous research shows that *Bacillus* spores can modulate the immune system by supressing the overexpression of pro-inflammatory cytokines and enhancing mucosal IgA levels in children with ARTIs caused by RSV and influenza virus^30,31^. Based on this, we predicted that LiveSpo Navax could have similar effects in children with pneumonia from viral and bacterial infections. We examined changes in key pro-inflammatory cytokines (IL-6, IL-8, and TNF-α) and mucosal Immunoglobulin A (IgA) in nasopharyngeal samples between day 0 and day 3 to assess these effects, as symptoms showed the most noticeable improvement during this period. After 3 days of treatment, the Navax group showed significant reductions in IL-6 (4.6-fold, *p* < 0.0001) and IL-8 (1.5-fold, *p* = 0.0094), whereas the Control group showed no significant reduction (*p* = 0.1491 and 0.6109, respectively) (Fig. 4a1, 4b1). Both groups experienced a marked decrease in TNF-α levels after 3 days (*p* < 0.0001). The Navax group showed an almost 600-fold reduction (from 47.94 to base line of 0.08 pg/mL), nearly 30-fold greater than the Control group (from 48.77 to 2.40 pg/mL) (Fig. 4c1). Additionally, we assessed the effect of *Bacillus* spore nasal spray on changes in IgA levels (µg/mL) in the nasopharynx, comparing measurements between days 0 and 3. Both groups exhibited a slight increase in IgA levels (Fig. 4d1) from day 0 to day 3, with the Navax group showing a more robust 1.7-fold increase, which was statistically significant (*p* = 0.038). These findings indicate that nasal-spraying *Bacillus* spores significantly reduced the overproduction of IL-6, IL-8, and TNF-α cytokines, and moderately increased nasal IgA levels in response to RSV.

Moreover, we used multiple logistic regression to evaluate the correlation between reduced RSV load and changes in cytokine levels and IgA concentrations (Figs. 4a2–d2). A reduction threshold of 10² was also maintained for RSV load, with cytokines/IgA set at zero. Patients with RSV reductions greater than 10² showed a significant decrease in IL-6 (*p* = 0.005), IL-8 (*p* = 0.01), and TNF-α (*p* = 0.01) levels in Navax group, as indicated by red plots distributing on the upper horizontal center line (Figs. 4a2–c2). Meanwhile, the Control group had more variable plots with some patients even showing increased cytokine levels. Additionally, most patients in the Navax group who experienced substantial RSV reduction also saw an increase in IgA levels, unlike the Control group, which showed no clear trend (*p* = 0.005, Fig. 4d2). Overall, the Navax group demonstrated a stronger reduction in viral load, which correlated with lower cytokine levels and higher IgA levels compared to the Control group.

### Improving nasal microbiota diversity and density at the phyla, genus and species levels by nasal-spraying *Bacillus* spores

**Fig. 5:**
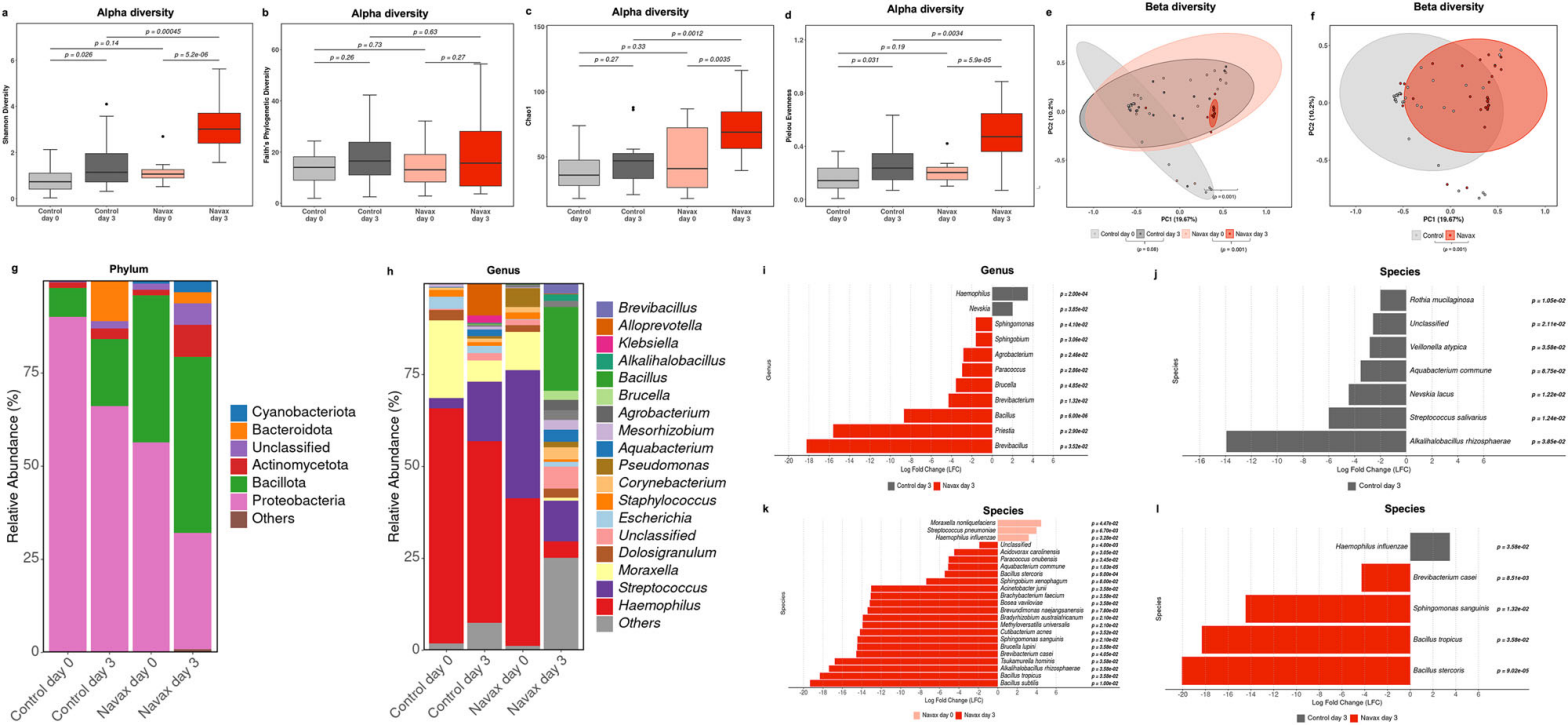
Impact of *Bacillus* spore nasal spray on the nasal microbiome in pediatric RSV pneumonia. Alpha diversity (Shannon, Chao1, Faith’s PD, Pielou Evenness) (**a**–**d**) and beta diversity (PCoA) (**e**, **f**) of 16S rRNA microbiota in nasopharyngeal samples from Control and Navax groups at day 3 compared to day 0. The Wilcoxon test and Mann–Whitney test were used for comparisons of alpha diversity within each group and between the two groups. The significance level for all analyses was set at *p* < 0.05. Distribution of 6 major phyla in the two groups at day 3 compared to day 0 (**g**). Distribution of the 18 major genera in the Navax and Control groups at day 3 compared to day 0 (**h**). Log_2_ fold change (LFC) in the relative abundance of genera in the Navax group compared to the Control group at day 3 (**i**). LFC in the relative abundance of species in the Control group at day 0 compared to day 3 (**j**)**;** in the Navax group at day 0 compared to day 3 (**k**); in the Navax group compared to the Control group at day 3 (**l**). The Lefse algorithm was used to evaluate the differences between these characteristic markers. Only markers with statistically significant differences (*p* < 0.05) were included in the analysis. Error bars in panels **a**–**d** indicate the upper extreme (Q3 + 1.5 × IQR) and the lower extreme (Q1 – 1.5 × IQR). Sample size: *n* = 16 biologically independent samples in the Control group and *n* = 16 in the Navax group.

The nasal microbiota plays a crucial role in respiratory health. A balanced nasal microbiota signals good health, while imbalances increase infection risk^38,39^. To evaluate probiotic effects, we analyzed nasal microbiota changes from 16 representative samples in each group between day 3 and day 0. Initially, both the Navax and Control groups had similar microbial diversity (Fig. 5a-d). After 3 days, diversity increased in both groups, with the Navax group showing more significant increases: 2.8-fold in Shannon (*p* = 5.2e-06), 1.7-fold in Chao1 (*p* = 0.0035), and 2.3-fold in Pielou’s evenness indices (*p* = 5.9e-05). The Control group exhibited smaller increases with 1.8-fold in Shannon (*p* = 0.026) and 1.7-fold in Pielou’s evenness (*p* = 0.031), while the increase in Chao1 was not significant (*p* = 0.27). Notably, the Navax group had a 1.3–1.8-fold higher diversity than the Control group on day 3 (Shannon: *p* = 0.0045; Chao 1: *p* = 0.0012; Pielou’s evenness: *p* = 0.0034). Data obtained from PCoA analysis revealed a more localized distribution in the Navax group on day 3 compared to broader distributions in the Control group (Fig. 5e). Overall, nasal microbiota composition differed significantly between the groups both before and after probiotic treatment (Fig. 5f). Furthermore, we found that the nasal microbiota composition in the analyzed groups was primarily dominated by bacteria from the phyla Proteobacteria, Bacillota (Firmicutes), and Actinobacteria, while Cyanobacteria, Bacteroidetes, and other phyla were present in significantly lower density. Before treatment, both groups had high densities of Proteobacteria. However, by day 3, Proteobacteria significantly decreased, more so in the Navax group (1.80-fold) than in the Control group (1.36-fold). Conversely, Bacillota increased by day 3, with 1.20-fold in the Navax group and 2.30-fold in the Control group (Fig. 5g).

In the genus-level taxonomic analysis, we evaluated the 18 genera with the highest relative abundances (Fig. 5h). At day 0, the microbiota structure of both the Navax and Control groups was similar, predominantly comprising of genera commonly associated with respiratory pathogens, with *Haemophilus* accounting for the highest proportion (40–64%), followed by *Moraxella* (10–21%) and *Streptococcus* (2–35%). Meanwhile, the relative abundance of beneficial genera such as *Bacillus, Brevibacillus and Priestia* was low (<0.3%) in both groups. No statistical difference in their abundance was observed between the Control vs. Navax groups. By day 3, in the Control group, *Haemophilus* and *Moraxella* decreased by only 1.29- and 3.65-fold, with relative abundances of 49.49% and 5.77%, respectively, while *Streptococcus* increased 5.44-fold to 16.14%. In contrast, in the Navax group, *Haemophilus, Moraxella*, and *Streptococcus* were significantly reduced by 9.08-, 13.33-, and 3.15-fold, reaching relative abundances of 4.42%, 2.12%, and 1.87% at day 3, respectively. Notably, the Navax group exhibited increased diversity of beneficial genera after 3 days, with the emergence of *Bacillus* (0.26% at day 0 vs. 22.91% at day 3), *Alkalihalobacillus* (not detectable at day 0 vs. 1.70% at day 3), and *Brevibacillus* (not detectable at day 0 vs. 2.89% at day 3). In contrast, these genera were almost absent in the Control group at both time points. In summary, the nasal microbiota composition in the Control and Navax groups was comparable on day 0, but showed distinct differences on day 3.

We further conducted a Lefse algorithm analysis^36^ to identify specific genera and species exhibiting changes in density, either between the two groups or between the two time points of probiotic intervention (days 0 and 3) within each group. Results shown in Fig. 5i indicated 11 genera having significant differences in densities between the two groups at day 3 of the treatment period. Most notable were indicators directly related to the respiratory tract, including *Haemophilus*, *Bacillus*, *Brevibacillus*, and *Priestia*. *Haemophilus* showed higher residual presence in the Control group vs. the Navax group with a Log_2_ Fold Change (LFC) of 3.49 (*p* = 0.0002). The Navax group retained the presence of *Brevibacillus* and *Priestia*, which were absent in the Control group (LFC ranging from −15.61 to −18.23). On the other hand, the *Bacillus* genus was present at more than an 8.67-fold higher level in the Navax group compared to the Control group.

In terms of species taxonomy, in the Control group, we observed an increase in only two nasal commensal species, *Streptococcus salivarius* and *Veillonella atypica* (LFC ranging from −2.84 to −6.01), and no reduction in the harmful species *H. influenzae* at day 3 compared to day 0 (Fig. 5j). As shown in Fig. 5k, there was a noticeable change in the species composition of the Navax group from day 0 to day 3. At day 0, the dominant co-infection bacterial species related to pneumonia in children, such as *M. nonliquefaciens*, *S. pneumoniae*, and *H. influenzae*, were significantly higher compared to day 3, with LFC (Navax day 0 vs. day 3) of 4.46 (*p* = 0.0447), 3.97 (*p* = 0.0067), and 3.18 (*p* = 0.0328). Conversely, by day 3, the Navax group exhibited a significant increase in beneficial species from the genera *Bacillus* and *Brevibacterium*, including *B. subtilis* (LFC = −19.34, *p* = 0.01), *B. tropicus* (LFC = −18.32, *p* = 0.0358), *B. casei* (LFC = −14.57, *p* = 0.0405), and *B. stercoris* (LFC = −5.47, *p* = 0.0009), which were almost absent at day 0 (Fig. 5k). Additionally, at day 3, the specific species indicator for the Control group was *H. influenzae* compared to the Navax group (LFC = 3.49, *p* = 0.0358). In contrast, the Navax group was distinguished from the Control group by the presence of beneficial species such as *B. tropicus* (LFC = −18.32, *p* = 0.0358), *B. stercoris* (LFC = −20.08, *p* = 9.02e−5), and *B. casei* (LFC = −4.29, *p* = 0.00851), which were nearly absent in the Control group after 3 days of treatment (Fig. 5l).

## Discussion

Pediatric community-acquired pneumonia (CAP) is associated with extended hospital stays, higher costs, and increased morbidity and mortality^40–42^. Probiotics offer a promising alternative for preventing and treating specific conditions^24,25^, with studies showing their anti-respiratory virus properties, making them a potential complementary treatment for viral respiratory infections.

Our study shows that nasal-spraying *Bacillus* spores (LiveSpo Navax) is safe and effective in supporting the treatment of pneumonia in children under two years old with RSV and bacterial co-infections, reducing treatment duration, hospital stays, and oxygen use. These findings reinforce our earlier research on RSV-induced ARTI, which primarily involved children with RSV bronchiolitis, a milder condition where only ~3.5% patients had SpO_2_ < 95% and supplemental oxygen was rarely needed^30^. In contrast, the current study focuses on the population of children with RSV pneumonia, a more severe condition, 77% of whom had SpO_2_ < 92% and required oxygen therapy, highlighting both the increased severity and the efficacy of *Bacillus* spores in this context. Compared to oral probiotics, our results show notably faster (days vs. months) and much higher efficacy (hundreds of times more effective) when used as adjunct therapy for ARTI^26–29,43–46^.

Voiriot et al. (2016) have reported that ICU patients with community-acquired pneumonia (CAP) and mixed bacterial-viral infections have a higher risk of hospital mortality and prolonged mechanical ventilation^47^. Our study showed that at day 3, the Navax group exhibited an effective reduction in both RSV and co-infected bacteria 16-fold greater than that in the Control group. This finding further supports our hypothesis that nasal-spraying *Bacillus* spores interact non-specifically with the mucosal immune system, viruses, and bacteria, potentially aiding in treating respiratory infections involving both viruses and bacteria. Moreover, the significant reduction in virus and bacterial burden by day 3 in the Navax group is particularly meaningful in reducing antibiotic use in bacterial treatment. Antibiotic treatment duration was shortened by 2 days, representing a 14.3% decrease in antibiotic usage. This absolute reduction was 1.5-fold higher than that reported by Hatakka et al. (2001) using *Lactobacillus* to treat respiratory tract infections in children attending preschool^43^.

In our study, we identified *H. influenzae* and *S. pneumoniae* as the predominant co-infecting bacteria in young children with RSV pneumonia, consistent with previous studies^48,49^. *H. influenzae* promotes inflammation and disease progression, whereas *S. pneumoniae* is associated with inflammatory effects^50^. Bacterial cell-wall components, like peptidoglycan and teichoic acid, activate TLR2, triggering transcriptional regulators such as nuclear factor kappa B (NF-κB). The cytokine response markedly influenced clinical progression and severity. IL-6 and TNF-α played critical roles in bacterial infections, while RSV infection was directly associated with IL-8, an important chemokine involved in neutrophil recruitment^51^. Probiotics positively regulate the host’s immune system, including innate and adaptive immunity, by adjusting the activities and functions of CD4 + T/CD8 + T cells, B cells, and dendritic cells (DCs)^31,52,53^. Our findings suggest that nasal-spraying *Bacillus* spores can modulate immune responses by acting through cellular intermediates, leading to significant reductions in elevated pro-inflammatory cytokine levels, including IL-6, IL-8, and TNF-α. In pneumonia, cytokine secretion varies considerably depending on the causative microorganism involved^54^. The greater reduction in IL-6 observed in our study may reflect the immunomodulatory effects of *Bacillus* spores, which are known to interact with immune cells such as dendritic cells and macrophages via Toll-like receptors (TLRs), leading to a balanced immune response. By targeting IL-6 overproduction, a key driver of inflammation in respiratory infections like RSV-related pneumonia^55^, *Bacillus* spores likely help prevent inflammation-induced tissue damage while maintaining effective pathogen defense. These findings align with prior reports and highlight the potential of *Bacillus* spores in mitigating hyperinflammatory responses in respiratory infections^56,57^.

The presence of bacteria in RSV pneumonia patients has been shown to increase viral loads and decrease specific antibodies such as IgA, IgM, and IgG^58–60^. IgA, the most abundant antibody isotype in the mucosal immune system, plays a critical role in neutralizing pathogens at mucosal surfaces^61^. Probiotics can enhance IgA production by activating Peyer’s patches, dendritic cells, macrophages, and Toll-like receptors, promoting adaptive immune responses^62,63^. Our findings align with these observations, showing that spraying *Bacillus* probiotics significantly enhanced IgA production while also inducing notable decreases in RSV load, co-infecting bacteria concentrations, and cytokine levels. Correlation analysis suggested that *Bacillus* spores could stimulate nasal mucosal IgA secretion, potentially aiding in general virus resistance and bacterial co-infections by modulating immune system hyperactivation^31^.

The human microbiota, a diverse mix of microorganisms, is essential for health and immunity. In addition to the digestive tract, these microorganisms are found in other areas, including the nasal cavity, where beneficial bacteria help prevent the colonization of opportunistic pathogens^64^. This direct nasal-spraying approach with liquid-form probiotics was designed to rapidly improve the nasal microbiota and enhance its ability to compete with bacteria that cause respiratory diseases. The method is based on the hypothesis that the microbiota plays a crucial role in maintaining lung immune functions and preventing respiratory illnesses^53,65^. Consistent with other studies, our study found that the dominant phyla were Proteobacteria, Bacillota, and Actinobacteria, with Cyanobacteriota, Bacteroidetes and other phyla presented at lower densities^65^. Nevertheless, our sample did not include the previously reported Fusobacteria phylum^39,66^, which may be due to nasal microbiota disorders linked to pneumonia, as sick children typically have less diverse nasal microbiota than healthy children^39,67^. The Navax group showed significant recovery in α and β diversity at day 3 compared to day 0 and the Control group, indicating that LiveSpo Navax substantially restored the diversity of the nasal microbiota in pneumonia patients. The increase in nasal microbiota diversity in the Control group at day 3 compared to day 0 agrees with prior studies indicating temporary microbiota dysbiosis during acute RSV infection and partial restoration afterward^68^. We hypothesize that broad-spectrum antibiotics reduced pathogenic bacteria such as *Haemophilus*, *Moraxella*, and *Streptococcus*, allowing less abundant taxa to regrow. In the Navax group, probiotics further enhanced diversity by more efficiently reducing these pathogens while promoting beneficial genera such as *Bacillus* and *Brevibacillus*. This finding was consistent with the markedly improved clinical and sub-clinical symptoms observed at day 3. Notably, we observed that the genus *Haemophilus* dominated on day 0 in both groups, consistent with the detection by real-time PCR and pathogenic mechanism involving the increase of *Haemophilus* in children infected with RSV^39,69^. After 3 days, the density of *Haemophilus* in the Navax group decreased about 8-fold more effectively than in the Control group on the same day, with *H. influenzae* being much higher in the Control group. Additionally, other harmful species including *M. nonliquefaciens*, *S. pneumoniae*, and *H. influenzae* in the Navax group were reduced by 10–20 folds compared to day 0. The balancing of the nasal microbiota in the Navax group on day 3 was also evident by the increase in beneficial species, such as *B. casei*, *B. subtilis*, *B. tropicus*, and *B. stercoris*, whereas the Control group only showed an increase in the beneficial bacterium *S. salivarius*. While oral probiotics are known to enhance gut microbiota and potentially reduce respiratory diseases via the gut-lung axis, there has been limited focus on their impact on nasal microbiota^53,70,71^. This study is, to the best of our knowledge, the first to demonstrate that direct nasal-spraying of *Bacillus* spores can enhance nasal microbiota, improving pneumonia symptoms and reducing antibiotic dependence.

This study had some limitations. First, due to ethical considerations in research involving young children, nasopharyngeal samples were collected only at two time points-day 0 and day 3-to minimize potential discomfort and distress for the participants. The small sample volumes obtained were sufficient for measuring viral load, bacterial co-infection, *Bacillus* sp., microbiome analysis, three cytokines, and IgA. However, this limited sampling prevented the assessment of other relevant pro-inflammatory cytokines and specific lymphocyte subpopulations linked to pneumonia. Second, due to budget constraints, only a third of the samples underwent 16S rRNA metagenomics, which may not fully capture the cohort’s diversity. Nevertheless, we believe that the assessments have provided several novel observations including (i) significant differences in alpha and beta diversity indices, and (ii) changes in the abundance of major genera and species. These findings should serve as a solid foundation for future studies with a larger sample size and longer follow-up periods, such as at patient discharge, to build on and expand these characterizations.

Based on our current study and previous reports^30,31^ we propose a model for the mechanism of action by which sprayed *Bacillus* spores alleviate RSV infection in the nasal tracts of children with RSV pneumonia through multiple pathways. The spores adsorb viral particles via non-specific interactions, neutralizing or inactivating the virus. By adhering to the nasal epithelium, the spores block binding sites for viruses and co-infecting bacteria, limiting their colonization^31,52,72^. Additionally, germinated spores produce antimicrobial compounds that inhibit bacterial growth. The spores also activate macrophages and dendritic cells, boosting nasal mucosal IgA production and recruiting cytotoxic T cells to clear infections. Modulating key pro-inflammatory cytokines (IL-6, IL-8, TNF-α), the spores can reduce excessive inflammation while maintaining immune defense, thereby lowering viral and bacterial loads^31,46,73^.

In conclusion, *Bacillus* spore nasal spray (LiveSpo Navax) appears to be a promising option for the treatment of bacterial superinfection in pediatric RSV pneumonia. To the best of our knowledge, this trial is the first to demonstrate the safety and efficacy of nasal-spraying *Bacillus* probiotics for treating pneumonia in children with RSV and bacterial co-infections. The approach reduced oxygen intervention by 2 days, shortened treatment by 1 day, and cut antibiotic duration by 1 day (14.3% reduction in duration of antibiotic treatment). After only 3 days of treatment, *Bacillus* spores reduced RSV and co-infected bacteria (*H. influenzae* and *S. pneumoniae*) by nearly 4000-fold, which was 16-fold more effective than standard treatment. In the Navax group, nasal pro-inflammatory cytokine levels of IL-6, IL-8, and TNF-α were significantly reduced by 4.6-, 1.5-, and nearly 600-fold, respectively, while IgA levels increased by 1.7-fold on day 3 compared to day 0. While metagenomic findings were based on a subset of samples, the results demonstrated improved microbial diversity, characterized by decreases in major harmful genera and species, e.g., *Haemophilus*, *Streptococcus*, and *Moraxella* and increases in key beneficial ones, such as *Bacillus, Brevibacillus*, and *Priestia*. In the era of increasing antibiotic resistance, our findings are of public health relevance and deserve further studies.

## Supplementary information

Supplementary Information

Description of Additional Supplementary files

Supplementary Data 1

Reporting Summary

## Data Availability

Supplementary Tables 1–2 and Supplementary Fig. 1 are provided in the Supplementary Information file (PDF). The dataset generated and analyzed during this study, containing de-identified demographic, clinical, and laboratory data underlying all figures and tables, is provided in the Supplementary data set file (Excel). Both the Supplementary Information (PDF) and Supplementary data set file (Excel) are accessible at: https://anabio.com.vn/documents/ (folder name: Supplementary data – Navax-RSV pneumonia). Additional de-identified individual participant data beyond the datasets shared are available from the corresponding author (vananhbiolab@gmail.com) upon reasonable request. Access will be granted for academic research purposes only, following approval by the Vietnam National Children’s Hospital.
