# Supplementary material for: Nasal-spraying Bacillus spore probiotics for pneumonia in children with respiratory syncytial virus and bacterial co-infections: a randomized clinical trial
Source: Commun Med (Lond). 2025 Aug 7;5:336. doi: 10.1038/s43856-025-01029-9 (PMC12328779; doi:10.1038/s43856-025-01029-9)
Supplement: Supplementary file 1 — Supplementary Information [file 43856_2025_1029_MOESM1_ESM.pdf]

Supplementary Information

Supplementary Table 1

Microbial culture-based detection of bacterial co-infection before treatment

**Names of bacterial co-infections**	**Control group (*N* = 50)**	**Navax group (*N* = 51)**	***p value***
	***n* (%)**	***n* (%)**	
H. influenzae	20 (40.00)	17 (33.33)	0.8309[a]
S. pneumoniae	4 (8.00)	6 (11.76)	
M. catarrhalis	5 (10.00)	4 (7.84)	
S. aureus	1 (2.00)	2 (3.92)	
P. aeruginosa	1 (2.00)	1 (1.96)	
H. influenzae + S. pneumoniae	2 (4.00)	1 (1.96)	
H. influenzae + M. catarrhalis	0 (0.00)	2 (3.92)	
**Total**	33 (66.00)	33 (64.71)	

[a] *Fisher's Exact test*

Supplementary Table 2

Distribution of cases requiring 1- or 2-type antibiotic treatment within the first 2 days

**Antibiotic therapy**	**Control** $N = 50$	**Navax** $N = 51$	***p-value***
1-type antibiotic treatment, n (%)	***41 (82.00)***	***43 (84.31)***	>0,9999 [a]
Ampicilin/sulbactam	4 (8.00)	4 (7.84)	
Cefotaxime	37 (74.00)	38 (74.51)	
Ceftriaxone	0 (0.00)	1 (1.96)	
2-type antibiotic treatment, n (%)	***9 (18.00)***	***8 (15.69)***	0.5765 [a]
Ampicillin/sulbactam+azithromycin	2 (4.00)	0 (0.00)	
Cefotaxime+azithromycin	6 (12.00)	6 (11.76)	
Cefotaxime+vancomycin	1 (2.00)	2 (3.92)	

[a] *Fisher's Exact test*

Supplementary Figure 1

Vital sign changes following nasal administration of LiveSpo Navax and physiological saline solution over 3 days

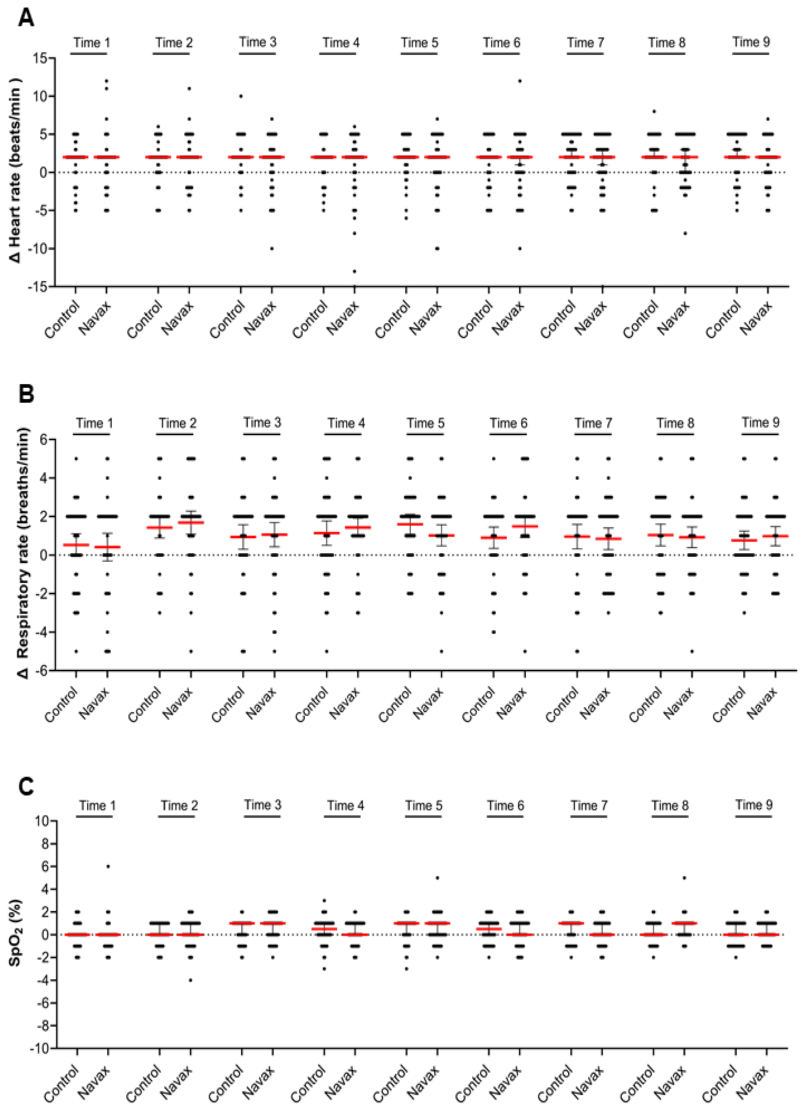

Vital signs of heart rate (A), respiratory rate (B), and SpO_2 (C) between before and after nasal-spraying with LiveSpo Navax (Navax group) and physiological saline solution (Control group), across 9 spraying times over 3 days. Graphs showing median values (red lines) lower-upper confidence limits (black lines), and measurements from individual patients (dots). Sample size: *n* = 50 biologically independent patients in the Control group and *n* = 51 in the Navax group.
