# Supplementary material for: Nasal-spraying Bacillus spore probiotics for pneumonia in children with respiratory syncytial virus and bacterial co-infections: a randomized clinical trial
Source: Commun Med (Lond). 2025 Aug 7;5:336. doi: 10.1038/s43856-025-01029-9 (PMC12328779; doi:10.1038/s43856-025-01029-9)
Supplement: Supplementary file 2 — Description of Additional Supplementary files [file 43856_2025_1029_MOESM2_ESM.pdf]

Description of Additional Supplementary files

File name: Supplementary Data 1

Description: Data set for all tables, figures, and supplementary information of the manuscript.
