# Supplementary material for: Nasal-spraying Bacillus spore probiotics for pneumonia in children with respiratory syncytial virus and bacterial co-infections: a randomized clinical trial
Source: Commun Med (Lond). 2025 Aug 7;5:336. doi: 10.1038/s43856-025-01029-9 (PMC12328779; doi:10.1038/s43856-025-01029-9)
Supplement: Supplementary file 4 — Reporting Summary [file 43856_2025_1029_MOESM4_ESM.pdf]

nature portfolio

Corresponding author(s): Anh Thi Van Nguyen

Last updated by author(s): 6/7/2025

Reporting Summary

Nature Portfolio wishes to improve the reproducibility of the work that we publish. This form provides structure for consistency and transparency in reporting. For further information on Nature Portfolio policies, see our Editorial Policies and the Editorial Policy Checklist.

Please do not complete any field with "not applicable" or n/a. Refer to the help text for what text to use if an item is not relevant to your study.
For final submission: please carefully check your responses for accuracy; you will not be able to make changes later.

Statistics

For all statistical analyses, confirm that the following items are present in the figure legend, table legend, main text, or Methods section.

n/a	Confirmed	
☐	☒	The exact sample size (n) for each experimental group/condition, given as a discrete number and unit of measurement
☐	☒	A statement on whether measurements were taken from distinct samples or whether the same sample was measured repeatedly
☐	☒	The statistical test(s) used AND whether they are one- or two-sided<br>*Only common tests should be described solely by name; describe more complex techniques in the Methods section.*
☐	☒	A description of all covariates tested
☐	☒	A description of any assumptions or corrections, such as tests of normality and adjustment for multiple comparisons
☐	☒	A full description of the statistical parameters including central tendency (e.g. means) or other basic estimates (e.g. regression coefficient) AND variation (e.g. standard deviation) or associated estimates of uncertainty (e.g. confidence intervals)
☐	☒	For null hypothesis testing, the test statistic (e.g. F, t, r) with confidence intervals, effect sizes, degrees of freedom and P value noted<br>*Give P values as exact values whenever suitable.*
☒	☐	For Bayesian analysis, information on the choice of priors and Markov chain Monte Carlo settings
☒	☐	For hierarchical and complex designs, identification of the appropriate level for tests and full reporting of outcomes
☒	☐	Estimates of effect sizes (e.g. Cohen's d, Pearson's r), indicating how they were calculated

Our web collection on statistics for biologists contains articles on many of the points above.

Software and code

Policy information about availability of computer code

Data collection	Data were collected by the project investigators and entered into Microsoft Excel.
Data analysis	Data analysis was performed using commercially available statistical software such as GraphPad Prism v8.4.3 and R package microbiome, as described in the Methods section. No custom software or code was developed in this study.

For manuscripts utilizing custom algorithms or software that are central to the research but not yet described in published literature, software must be made available to editors and reviewers. We strongly encourage code deposition in a community repository (e.g. GitHub). See the Nature Portfolio guidelines for submitting code & software for further information.

Data

Policy information about availability of data

All manuscripts must include a data availability statement. This statement should provide the following information, where applicable:

- Accession codes, unique identifiers, or web links for publicly available datasets
- A description of any restrictions on data availability
- For clinical datasets or third party data, please ensure that the statement adheres to our policy

Supplementary Tables 1–2 and Supplementary Figure 1 are provided in the Supplementary Information file (PDF). The dataset generated and analyzed during this study, containing de-identified demographic, clinical, and laboratory data underlying all figures and tables, is provided in the Supplementary data set file (Excel). Both the Supplementary Information (PDF) and Supplementary data set file (Excel) are accessible at: https://anabio.com.vn/documents/ (folder name: Supplementary data – Navax-RSV pneumonia). Additional de-identified individual participant data beyond the datasets shared are available from the corresponding author (vananhbiolab@gmail.com) upon reasonable request. Access will be granted for academic research purposes only, following approval by the Vietnam National Children’s Hospital.

Research involving human participants, their data, or biological material

Policy information about studies with human participants or human data. See also policy information about sex, gender (identity/presentation), and sexual orientation and race, ethnicity and racism.

Reporting on sex and gender	The study's patients include both male and female, as described in the Methods section and Table 1.
Reporting on race, ethnicity, or other socially relevant groupings	There were no reports of racial discrimination in this study.
Population characteristics	Research conducted in children from 1 to 24 months of age with pneumonia meeting the inclusion criteria shown in the Methods section.
Recruitment	Recruitment time (from July 2023 to March 2024) and recruitment criteria (inclusion and exculsion) are described in details in the Methods section and Fig. 1.
Ethics oversight	This study received ethics approval from the Ethics Committee in Medical Research of the Vietnam National Children's Hospital under Decision No.1241/BVNTW-HDDD on May 23, 2023. The research conducted to the ethical principles outlined in the Helsinki statement, the ICH GCP guidelines, and the prevailing ethical regulations and standards established by the Vietnam Ministry of Health for research involving human subjects. All of this information is described in the Methods section.

Note that full information on the approval of the study protocol must also be provided in the manuscript.

Field-specific reporting

Please select the one below that is the best fit for your research. If you are not sure, read the appropriate sections before making your selection.

☒ Life sciences ☐ Behavioural & social sciences ☐ Ecological, evolutionary & environmental sciences

For a reference copy of the document with all sections, see nature.com/documents/nr-reporting-summary-flat.pdf

Life sciences study design

All studies must disclose on these points even when the disclosure is negative.

Sample size	Sample size calculations were performed using the sample size calculator https://clincalc.com/Stats/SampleSize.aspx A description of expectations and sample size parameters is provided in the Methods section. Sample size was 120 (60 per group). This information is described in the Methods section.
Data exclusions	Patients excluded from the study were not included in the analysis. For ELISA assay of IL-8 and IgA, only measurable samples at day 0 were included in the statistical analysis. This information is described in the Methods section.
Replication	DNA/RNA extraction was performed twice to obtain 100 µL of purified DNA/RNA for downstream real-time PCR assays. However, each independent biological sample was measured only once in all assays, including real-time PCR, ELISA, 16S rRNA NGS for nasal microbiota analysis. This information is described in the Methods section.
Randomization	This is a randomized, controlled clinical trial. Participants were randomly assigned in a 1:1 ratio to receive standard care plus either LiveSpo Navax (Navax group) or physiological saline solution (Control group), using a simple sealed-number draw at enrollment. For nasal microbiome analysis, the nasopharyngeal samples were randomly selected in a stratified manner to ensure similar indices regarding age, sex, weight, pre-hospital illness, and median reduction in RSV after 3 days of intervention. This information is described in the Methods section.
Blinding	This study is a double-blind clinical trial. Upon obtaining informed consent from the children's parents, the chief nurse randomly selected sealed-paper coded numbers 1 or 2 from a carton box containing equal numbers of each code and immediately assigned these numbers to the participants. Product bottles were labeled with codes plus patient ID, along with patient name, date of birth, and room number to avoid confusion. Confidentiality was maintained throughout the study. Group allocations remained blinded to all personnel involved in patient care, treatment administration, and laboratory analyses. Operational adjustments were managed using anonymized codes, ensuring that the double-blind design was strictly preserved. The taste and smell of LiveSpo Navax (intervention product) were indistinguishable from those of physiological saline solution (control product).

Reporting for specific materials, systems and methods

We require information from authors about some types of materials, experimental systems and methods used in many studies. Here, indicate whether each material, system or method listed is relevant to your study. If you are not sure if a list item applies to your research, read the appropriate section before selecting a response.

Materials & experimental systems

n/a	Involved in the study
☐	☒ Antibodies
☒	☐ Eukaryotic cell lines
☒	☐ Palaeontology and archaeology
☒	☐ Animals and other organisms
☐	☒ Clinical data
☒	☐ Dual use research of concern
☒	☐ Plants

Methods

n/a	Involved in the study
☒	☐ ChIP-seq
☒	☐ Flow cytometry
☒	☐ MRI-based neuroimaging

Antibodies

Antibodies used	All antibodies used in this study to measure cytokines IL-6, IL-8, TNF-α, and IgA concentrations were provided as commercial ELISA kits by Invitrogen/Thermo Fisher Scientific (MA, US) and R&D Systems (MN, US) and are described in the Methods section. No “in-house” antiboties were used in this study.
Validation	All ELISA kits for IL-6 (Invitrogen/Thermo Fisher Scientific, Cat. No. 88-7066-76), IL-8 (R&D Systems, Cat. No. DY208), TNF-α (Invitrogen/Thermo Fisher Scientific, Cat. No. 88-7346-76), and IgA (Invitrogen/Thermo Fisher Scientific, Cat. No. 88-50600) were validated and used strictly according to the manufacturers’ protocols. No additional “in-house” validation was performed.

Clinical data

Policy information about clinical studies

All manuscripts should comply with the ICMJE guidelines for publication of clinical research and a completed CONSORT checklist must be included with all submissions.

Clinical trial registration	The study was registered with ClinicalTrials.gov, US National Library of Medicine (Identifier No: NCT05929599) on 30/06/2023. This information is presented in the Abstract and Methods section.
Study protocol	A full study protocol has been submitted online as a related file with the manuscript.
Data collection	Clinical data were collected as part of a double-blind, randomized, controlled clinical trial conducted at the Center for Pulmonology and Respiratory Care, Vietnam National Children’s Hospital, from July 2023 to July 2024. Patient enrollment occurred between July 2023 and March 2024. Data collection complied with ICMJE guidelines, and a CONSORT checklist is included with this submission.
Outcomes	Primary outcomes: median treatment duration for pneumonia symptoms (rhinitis, fever, retractions, rales, wheezy, cough, diarrhea, vomiting), oxygen therapy duration, and total treatment days.<br>Secondary outcomes: fold reduction of RSV and bacterial co-infections (S. pneumoniae, H. influenzae), nasal cytokine (IL-6, IL-8, TNF-α) and IgA levels, and microbiota composition, all at day 3 vs. day 0. All primary and secondary outcomes are clearly described in the Methods section.

This checklist template is licensed under a Creative Commons Attribution 4.0 International License, which permits use, sharing, adaptation, distribution and reproduction in any medium or format, as long as you give appropriate credit to the original author(s) and the source, provide a link to the Creative Commons license, and indicate if changes were made. The images or other third party material in this article are included in the article's Creative Commons license, unless indicated otherwise in a credit line to the material. If material is not included in the article's Creative Commons license and your intended use is not permitted by statutory regulation or exceeds the permitted use, you will need to obtain permission directly from the copyright holder. To view a copy of this license, visit http://creativecommons.org/licenses/by/4.0/
